# Clinical significance of granule‐containing myeloma cells in patients with newly diagnosed multiple myeloma

**DOI:** 10.1002/cam4.875

**Published:** 2016-10-13

**Authors:** Kazuhito Suzuki, Shingo Yano, Kaichi Nishiwaki, Koji Sano, Takaki Shimada, Yuichi Yahagi, Yoji Ogasawara, Katsuki Sugiyama, Shinobu Takahara, Takeshi Saito, Kinuyo Kasama, Jiro Minami, Hiroki Yokoyama, Yutaro Kamiyama, Atsushi Katsube, Hidekazu Masuoka, Mitsuji Katori, Tomohito Machishima, Aya Ouchi, Nobuaki Dobashi, Ken Kaito, Noriko Usui, Keisuke Aiba

**Affiliations:** ^1^ Division of Clinical Oncology/HematologyDepartment of Internal MedicineThe Jikei University School of MedicineTokyoJapan; ^2^ Central Clinical LaboratoryThe Jikei University HospitalTokyoJapan; ^3^ Division of Transfusion MedicineThe Jikei University School of MedicineTokyoJapan

**Keywords:** CD49e, CD56, granules, morphology, myeloma, prognosis

## Abstract

The clinical features and prognostic significance of myeloma cells containing granules remain unclear. The purpose of this retrospective study was to investigate the clinical significance of granule‐containing myeloma cells in patients with newly diagnosed multiple myeloma (NDMM). We retrospectively analyzed the records of 122 patients diagnosed with NDMM between January 2007 and December 2013. Granule‐containing myeloma cells were defined as myeloma cells that exhibited three or more granules in their cytoplasm by May‐Giemsa staining. The patients were classified into two groups, the granule‐containing myeloma (GM) and nongranule‐containing myeloma (non‐GM) groups, depending on the proportion of myeloma cells that contained granules (cut‐off value: 10%). There were 25 (20.5%) patients in the GM group. Patients in the GM group displayed significantly higher CD56 and CD49e expression than those in the non‐GM group (*t*‐test, *P *=* *0.027 and 0.042). None of the patient characteristics differed significantly between the two groups. There was no significant difference in the chemotherapy profiles of the two groups, and the overall response rates of the two groups were similar. During the median follow‐up period of 33.9 months, the overall survival (OS) in the GM group was similar to that in the non‐GM group; 4‐year OS of the GM and non‐GM groups were 78.5% and 51.9%, respectively (*P *=* *0.126). We concluded that cases of NDMM involving granule‐containing myeloma cells are not infrequent. Moreover, CD56 and CD49e expression was significantly higher in the presence of myeloma cell populations, and the presence of granules did not affect survival.

## Introduction

Multiple myeloma comprises a heterogeneous group of plasma cell neoplasms, which vary in terms of their morphology, phenotype, molecular biology, and clinical behavior. Even though the development of novel agents, such as bortezomib, thalidomide, and lenalidomide, has improved the prognosis of the condition over the last decade, multiple myeloma remains incurable because of its heterogeneity. Studies on multiple myeloma have identified a large number of prognostic factors for survival, which include staging the disease according to the international staging system (ISS) 1 and/or Durie‐Salmon staging system 2, detection of high‐risk cytogenetic abnormalities using fluorescence in situ hybridization (FISH) 3, 4, 5, 6, 7, plasma cell labeling index 8, presence of circulating plasma cells 9, and the patient's gene expression profile 10, 11, 12. Morphological findings also help in predicting the prognosis of multiple myeloma as in the case of other hematological malignancies. Greipp et al. 13 developed a morphological classification of myeloma cells. The plasmablastic group demonstrated significantly shorter survival rates than those with mature, intermediate, and immature cells.

Granules are detected in the cytoplasm of myeloma cells by May‐Giemsa staining in some cases. Since the first identification of granule‐containing myeloma cells by Steinmann, several case reports about granule‐containing myeloma have been published 14, 15, 16. However, the clinical features of granule‐containing myeloma cells have not yet been elucidated. The purpose of this retrospective study was to investigate the clinical significance of granule‐containing myeloma cells, in terms of patient characteristics, laboratory results, morphological findings, and prognosis, in patients with newly diagnosed multiple myeloma (NDMM).

## Materials and Methods

We reviewed the medical records of NDMM patients at Jikei University Hospital or Jikei Kashiwa Hospital diagnosed between January 2007 and October 2013 and were followed up until December 2014. This study was approved by the independent ethics committee/institutional review board of our institution.

### Patients

Patients were included if they had symptomatic multiple myeloma and were older than 20 years. Symptomatic multiple myeloma was defined as serum monoclonal protein level of 3 g/dL or more and when 10% or more of the bone marrow plasma indicated any of the following conditions: calcium elevation, renal insufficiency, anemia, or bone disease (CRAB) 17, 18. The CRAB components were defined as follows; calcium elevation: serum calcium level >11.0 mg/dL, renal insufficiency: serum creatinine level >2 mg/dL, anemia: hemoglobin concentration <10 or 2 g/dL less than the lower limit of the normal range, and bone disease: lytic or osteopenic bone disease 19. Patients with monoclonal gammopathy of undetermined significance (MGUS), smoldering multiple myeloma, or primary plasma cell leukemia were excluded from the analysis.

### Analysis of morphological findings

Granule‐containing myeloma cells were defined as myeloma cells in which three or more granules were detected in the cytoplasm by May‐Giemsa staining. The ultrastructure of typical granule‐containing myeloma cells was analyzed by electron microscopy. Granule‐containing myeloma cells were identified after differential counts of 200 cells had been performed by three hematologists and two laboratory technicians. Three individuals, including two hematologists and one laboratory technician, identified the granule‐containing myeloma cells in each hospital. Each individual counted 200 cells of each of the patients in the hospital in which they worked. Based on the percentage of the myeloma cells that contained granules (cut‐off value: 10%), the patients were classified into two groups; the granule‐containing myeloma group (GM) and the nongranule‐containing myeloma group (non‐GM). The patients' morphological findings were evaluated using the Greipp criteria, such as mature subtype, intermediate subtype, immature subtype, and plasmablastic subtype 13.

### Treatment and response assessment

Of the 122 patients, 112 patients received standard induction therapy regimens, such as bortezomib plus dexamethasone (BD); melphalan, bortezomib, and prednisolone (MBP); vincristine, adriamycin, and dexamethasone (VAD); melphalan plus prednisolone (MP); or high‐dose dexamethasone (HDD). Seventeen patients received high‐dose melphalan followed by autologous peripheral blood stem cell transplantation after induction therapy. Patients who relapsed or had refractory disease received salvage therapy involving BD and MBP; cyclophosphamide, bortezomib, and dexamethasone (CVD); bortezomib, thalidomide, and dexamethasone (BTD); bortezomib, lenalidomide, and dexamethasone (BLD); lenalidomide plus low‐dose dexamethasone (Ld); thalidomide plus dexamethasone (TD); ranimustine, vincristine, melphalan, and dexamethasone (ROAD) 20; MP; HDD; or VAD. Disease response was assessed according to the International Myeloma Working Group criteria 21.

### Prognostic factors

The following parameters were recorded and evaluated in each myeloma group: age, gender, hemoglobin concentration, estimated glomerular filtration rate (eGFR), serum C‐reactive protein (CRP) level, serum lactate dehydrogenase (LDH) level, serum beta‐2 microglobulin (*β*2m) level, disease stage according to the ISS, morphological subtype according to Greipp's criteria, surface antigen expression patterns analyzed by flow cytometry (FCM), and cytogenetic abnormalities. FCM analysis was performed in a single laboratory at Special Reference Laboratories, Inc. (SRL, Tokyo Japan) using antibodies against CD19, CD33, CD45, CD49e, and CD56. The expression levels of CD19, CD33, CD45, CD49e, and CD56 in CD38‐positive cells were evaluated using a two‐color panel of antibodies. Cytogenetic analyses were performed using Q banding karyotyping at Jikei University Hospital and G banding karyotyping at Jikei Kashiwa Hospital.

### Statistical analysis

Overall survival (OS) was calculated from the date of diagnosis until death from any causes or the last follow‐up. The time to next treatment (TTNT) was calculated from the date of diagnosis to the date when second‐line treatment was started. Fisher's exact test was used to compare various parameters between the GM group and the non‐GM group. Actuarial survival analysis was performed using the Kaplan–Meier method, and the resultant curves were compared using the log‐rank test. The correlation between the number of granules and the ratio of GM versus non‐GM cells was analyzed by Pearson's product‐moment correlation coefficient. All reported p‐values are two‐sided, and *P*‐values <0.05 were considered to be statistically significant. All statistical analyses were performed with EZR (Saitama Medical Center, Jichi Medical University), which is a graphical user interface for R (The R Foundation for Statistical Computing) 22. More precisely, it is a modified version of R Commander that adds frequently used biostatistical functions.

## Results

### Patients and morphological findings

**Figure 1:**
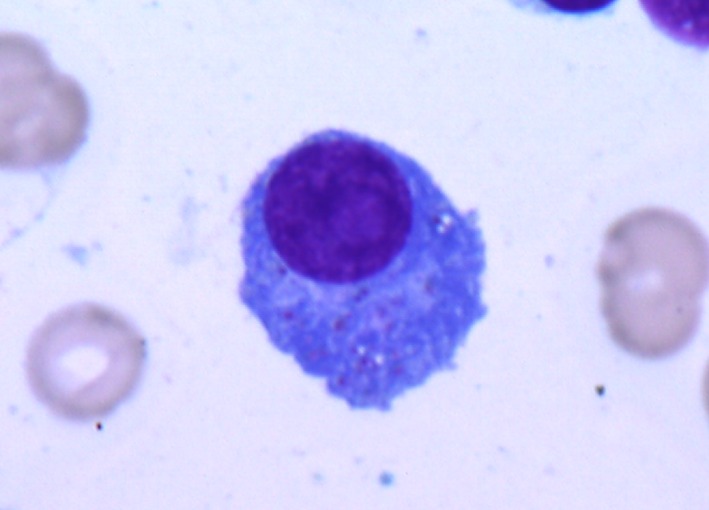
Morphological findings of a typical granule‐containing myeloma cell. Two myeloma cells had numerous azurophilic granules in cytoplasm. The granule‐containing myeloma cells were not positive by periodic acid‐schiff staining, and alpha‐naphthyl acetate esterase staining (not shown).

**Figure 2:**
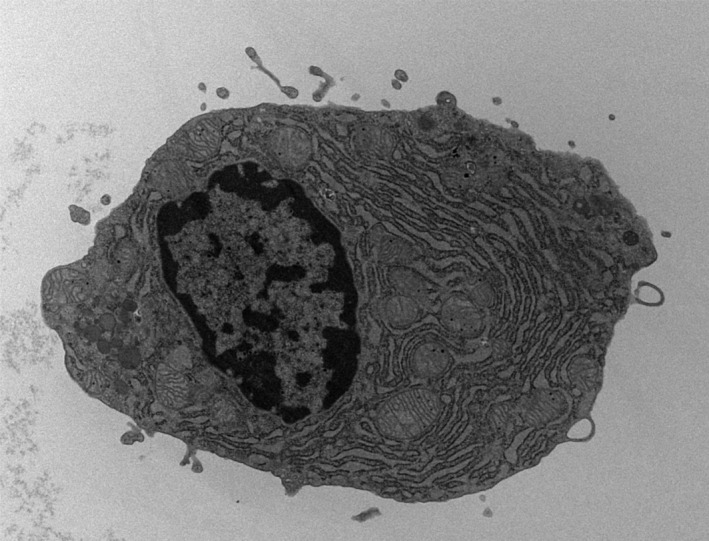
Electron microscopic findings of a typical granule‐containing myeloma cell. Several cytoplasmic inclusions with uniform color tone were pointed out.

One hundred and twenty‐two patients were diagnosed with symptomatic multiple myeloma between January 2007 and October 2013. The patient characteristics are shown in Table 1. The median age of the patients was 68 years (range: 37–89). Twenty‐five (20.5%) patients were assigned to the GM group. The morphological findings of a typical patient are shown in Figure 1. Nine bone marrow samples of the patient were evaluated at the time of diagnosis using myeloperoxidase (MPO), Periodic Acid‐Schiff (PAS), and alpha‐naphthyl acetate esterase (EST) staining. All the samples were negative for myeloperoxidase and periodic acid‐schiff staining while four of the nine samples were positive in alpha‐naphthyl acetate esterase staining. The electron microscopic findings of a GM cell from a typical patient are shown in Figure 2. In the GM group, the mean and median proportions of granule‐containing myeloma cells were 29.3% and 28% (range, 10–87%), respectively. The average and median numbers of granules in a granule‐containing myeloma cell were 6.0 and 5.8 (range 3–16), respectively. There was no significant difference between the number of granules and proportion of granule‐containing myeloma cells by Pearson's product‐moment correlation coefficient (*r *=* *0.299, 95% CI, −0.165 to 0.655, *P *=* *0.200). No significant differences in the hemoglobin concentration, disease risk according to cytogenetic abnormalities, frequency of each M‐protein subtype, ISS disease stage, eGFR, or serum levels of LDH, CRP, creatinine, albumin, or calcium were detected between the two groups at the time of diagnosis and were age and gender independent. The frequency of each morphological subtype according to the Greipp criteria was as follows: mature subtype, 47 patients; intermediate subtype, 46 patients; immature subtype, 25 patients and plasmablastic subtype, four patients. None of the morphological findings exhibited significant association with the GM group.

**Table 1:** Patient characteristics

	All patients	Granule myeloma (*n* = 25)	Nongranule myeloma (*n* = 97)	*P* value
Age				
Median	68 years (37–89)			
≤65 years	50	10	40	0.999
>65 years	72	15	57	
Gender				
Male	63	15	48	0.378
Female	59	10	49	
M‐protein subtype				
IgG	76	19	57	0.164
IgA	24	4	20	
BJP	15	2	13	
Others	7	0	7	
Light chain type				
Kappa chain	57	12	45	0.811
Lambda chain	48	9	39	
NA	17	4	13	
Cytogenetic abnormality				
Yes	31	10	21	0.118
No	76	13	63	
NA	15	2	13	
International staging system				
3	35	11	24	0.161
2	47	7	40	
1	31	5	26	
NA	9	2	7	
Positive	59	12	47	0.999
Negative	63	13	50	
eGFR				
≥50 mL/min	57	9	48	0.602
<50 mL/min	43	9	34	
NA	22	7	15	
Serum level of LDH				
>UNL	29	6	23	0.999
≤UNL	92	19	73	
NA	1	0	1	
Serum level of CRP				
>UNL	43	12	31	0.164
≤UNL	88	13	65	
NA	1	0	1	
Bortezomib containing induction therapy				
Yes	37	5	32	0.317
BD	23	3	20	
MPB	13	2	11	
CBD	1	0	1	
No	85	20	65	
MP	45	11	34	
VAD	17	3	14	
HDD	7	0	7	
Other	6	3	3	
None	10	3	7	
Autologous stem cell transplant				
Yes	18	1	17	0.191
No	104	24	80	
Immunomodulatory drugs containing chemotherapy as salvage therapy				
Yes	53	14	39	0.1
No	59	8	51	
Not received salvage therapy	10	3	7	

BJP, Bence Jones protein; ISS, International Staging System; CRAB, calcium elevation, renal insufficiency, anemia and bone disease; LDH, lactase dehydrogenase; CRP, C‐reactive protein; *β*2m, beta2 microglobulin; BD, bortezomib plus dexamethasone; MPB, melphalan, predonisolone plus bortezomib; CBD, cyclophosphamide, bortezomib plus dexamethasone; MP, melphalan plus predonisolone; VAD, vincristine, adriamycin plus dexamethasone; HDD, high‐dose dexamethasone; UNL, upper normal limit; NA, not available.

The expression of each surface antigen in the GM and non‐GM groups is shown in Table 2. The expression of CD56 and CD49e were significantly higher in the GM group than in the non‐GM group (76.5% vs. 55.9%, *P = *0.027, and 11.3% vs. 6.8%, *P = *0.042). The expression of CD19, CD33, and CD45 did not differ significantly between the GM and non‐GM groups.

**Table 2:** Antigens in granule myeloma cells and nongranule myeloma cells

Antigens	Expression level ± SD		*P* value
	Granule myeloma group	Nongranule myeloma group	
CD19	5.46 ± 12.88	5.19 ± 8.41	0.904
CD33	18.36 ± 19.29	17.85 ± 21.84	0.928
CD45	18.465 ± 21.06	24.46 ± 25.07	0.299
CD49e	11.34 ± 11.91	6.81 ± 8.42	0.042
CD56	76.54 ± 33.63	55.85 ± 40.55	0.027

SD; standard deviation, CD; cluster of differentiation.

### Response and survival

Of the 122 patients, 112 received induction therapy. The patients' treatment profiles are shown in Table 1. The proportion of patients who received bortezomib‐based induction therapy was similar in the GM and non‐GM groups (22.7% vs. 35.5%, *P *=* *0.317). There were no significant differences in the numbers of patients who received autologous stem cell transplant, or salvage chemotherapy combined with immunomodulatory drugs between the GM and non‐GM groups (4.8% vs. 23.3%, *P *=* *0.191, and 63.6% vs. 43.3%, *P = *0.100). The responses to chemotherapy in the GM and non‐GM groups are shown in Table 3. Twenty‐five patients achieved very good partial response (VGPR) or better; thirty patients achieved partial response (PR); 44 patients exhibited stable disease (SD); and five patients had progressive disease (PD). Seven patients could not be evaluated, including discontinuation of therapy in six patients because of adverse events (one case each of infection, renal failure, peripheral neuropathy, respiratory failure, dehydration, and myasthenia gravis) and missing data for one patient. The VGPR or better and overall response rate were 22.7% and 50.0%, respectively. The VGPR or better rate of the GM group was similar to that of the non‐GM group; 14.3% versus 24.7% (*P *=* *0.394). The overall response rate was also similar between the two groups; 38.1% in GM versus 52.8% in non‐GM (*P *=* *0.332).

**Table 3:** Response to initial chemotherapy

Response	Number of patients (ratio)		*P* value
	Granule myeloma group	Nongranule myeloma group	
ORR	38.1% (8/21)	52.8% (47/89)	0.332
VGPR ratio	14.3% (3/21)	24.7% (22/89)	0.394
VGPR or better	3	22	
PR	5	25	
SD	10	34	
PD	1	5	
Discontinuation of AEs	2	4	
	1 renal failure	1 respiratory failure	
	1 peripheral neuropathy	1 infection1 dehydration1 myasthenia gravis	
		
		
NA	1	0	

ORR, overall response rate; VGPR very good partial response; PR, Partial response; SD, stable disease; PD, progressive disease; AEs, adverse events; and NA, not available.

**Figure 3:**
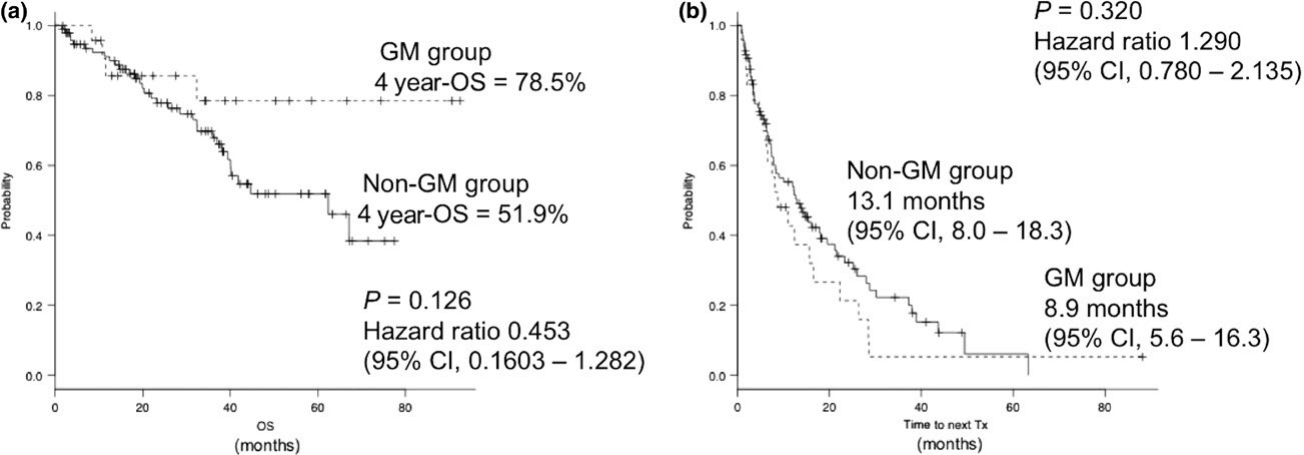
(A) Overall survival in the GM group and non‐GM groups. The 4‐year OS of the GM and non‐GM groups were 78.5% and 51.9%, respectively (*P *=* *0.126, HR: 0.453, 95% CI: 0.1603–1.282). (B) Time to next treatment in the GM and non‐GM groups. The median TTNT in the GM group was similar to that in the non‐GM group (8.9 vs. 13.1 months, *P *=* *0.320, HR: 1.290, 95% CI: 0.780–2.135).

The median follow‐up period for survival patients was 33.9 months. The 4‐year OS of the GM and non‐GM groups were 78.5% and 51.9%, respectively (*P *=* *0.126, HR: 0.453, 95% CI: 0.1603–1.282, Fig. 3A). There were no significant differences in OS between the patients treated with or without bortezomib as initial chemotherapy in both the GM and non‐GM groups; the 4‐year OS of the GM and non‐GM groups treated with bortezomib were 75.0% versus 54.1% (*P *=* *0.66) and those treated without bortezomib 79.9% versus 54.9% (*P *=* *0.159), respectively. The median TTNT in the GM group was similar to that in the non‐GM group (8.9 months vs. 13.1 months, *P *=* *0.320, HR: 1.290, 95% CI: 0.780–2.135, Fig. 3B). There were no significant differences in TTNT between the patients treated with or without bortezomib as initial chemotherapy in both the GM and non‐GM groups; median TTNT of the GM and non‐GM groups treated with bortezomib was 6.3 months versus 11.5 months (*P *=* *0.0576) and in those treated without bortezomib, was 12.6 months versus 12.8 months (*P *=* *0.605), respectively.

The CD56 and CD49e expression in the GM group were higher than those in the non‐GM group as mentioned earlier. We evaluated OS and TTNT in the CD56‐positive and negative groups, and CD49e‐positive and negative groups. There were no significant differences in OS and TTNT between the CD56‐positive and negative groups (*P *=* *0.705 and 0.717). There were also no significant differences in OS and TTNT between the CD49e‐positive and negative groups (*P *=* *0.860 and 0.890). Thus, the CD56 and CD49e expressions were not significantly different between the GM and non‐GM groups and the expression of CD56 and CD49e had no significant impact on survival.

## Discussion

The significance of the presence of cytoplasmic granules in myeloma cells is poorly understood. The GM cells are considered to be detectable infrequently because the study about large number of cases with the GM cells has not been demonstrated, and only several cases with cytoplasmic granules in myeloma cells were reported 14, 16, 23. In our study, 20.5% of MM patients displayed cytoplasmic granules, and hence we speculated that GM cells could be detected occasionally. There is no established definition of GM cell because earlier reports suggested that patients with GM cells were rare. We defined the criteria of the GM cells as below: the lower limit for the number of granules was set at three, similar to the criterion for large granular lymphocytes, which had three or more granules in the cytoplasm. The cut‐off value for classification of the disease as GM was 10% or more granule‐containing cells, similar to that as reported by Kurabayashi et al. 24 who demonstrated that the electron microscopic detection of several ultrastructural abnormalities in the cytoplasm of myeloma cells was a predictor of survival. The origin of the granules was controversial. Metzgerroth et al. 25 reported that the origin of these inclusions was lysosomal. In our study, the granules in the GM group were not related to the maturity of myeloma cells. The cells stained positive by EST in four of nine cases and none were positive in MPO or PAS staining. There are other reports, which demonstrated that Auer rod‐like inclusions were detected in cytoplasm of myeloma cells 25, 26, 27, 28, 29, 30, 31. We considered that the inclusions in the cells of the GM group were not identifiable with Auer‐like rods, because the shape of inclusions in the GM cells was quite different from that of Auer‐like rods. Finally, Auer‐like rods might be less frequent than granules because Auer‐like rods were not detected in our cases.

The CD56 and CD49e expression were significantly higher in the GM group compared to the non‐GM group. CD56 is a neural cell adhesion molecule that mediates cell–cell and cell–matrix interactions 32, and is expressed on myeloma cells in 70–80% of the myeloma patients 33, 34, 35, 36. CD56 is expressed at much higher levels in myeloma patients with osteolytic lesions. We analyzed the relationship between the occurrence of granules and bone lesions, which were detected using CT scan. The extent of bone lesions in the GM group was similar to that in the non‐GM group (54.2% vs. 68.1%, *P *=* *0.232). Absence of CD56 expression on myeloma cells was found to be associated with higher levels of *β*2m, Bence Jones proteinuria, renal insufficiency, thrombocytopenia, plasmablastic cell morphology, and shorter OS in 70 multiple myeloma patients who were treated with conventional chemotherapy 33. However, Mateo et al. and Hundemer et al. reported that conventional chemotherapy followed by autologous stem cell transplantation overcame the negative impact of low CD56 expression 37, 38. Serum *β*2m level, myeloma protein (M‐protein) subtypes, eGFR, platelet count, and morphological subtype were similar in CD56‐positive and negative groups in our study. We evaluated the patient characteristics such as complete blood count, urinary protein level, and serum levels of LDH and CRP in CD56‐positive and negative groups. These characteristics were also comparable between the CD56‐positive and negative groups. In addition, there was no significant difference of OS and TTNT between the patients in CD56‐positive and negative groups (data was not shown).

Our study also detected a significant relationship between the GM myeloma cells and the CD49e expression. CD49e is very late activation antigen‐5 (VLA‐5), which is a member of a family of heterodimeric transmembrane proteins that belong to the integrin family and plays an important role as a cell adhesion molecule 31. Kawano et al. 39 reported that VLA‐5‐negative myeloma cells were proliferative, IL‐6‐responsive immature cells. In contrast, VLA‐5‐positive myeloma cells were non proliferative, mature myeloma cells, non‐responsive to IL‐6 and secreted higher amounts of M‐protein than VLA‐5‐negative myeloma cells. The patient characteristics were similar in CD49e‐positive and negative groups. The biological significance of CD49e expression in the GM group was not clear and will be evaluated in our future studies.

There were two limitations to this study. First, only nine patient samples were evaluated by EST, MPO, and PAS staining. All these nine samples were collected from patients in the GM group at the time of myeloma diagnosis. We did not stain the samples from sixteen of the GM group patients by EST, MPO, and PAS because saved samples might not stain well compared with fresh samples just after bone marrow was collected. Therefore, it was not certain that the GM cell was stained by EST but not MPO and PAS. Second, the initial treatment might not have been suitable in the present time. BLD was one of the standard care methods as initial treatment across the world as the SWOG S0777 trial and the IFM/DFCI 2009 trial demonstrated 40, 41. However, lenalidomide as initial treatment was available in December 2015 in Japan. Therefore, no patients in this study received BLD as initial treatment. The clinical significance of granule‐containing myeloma cells might be different if majority of the patients were treated with BLD as first‐line therapy.

In conclusion, we found that patients with the GM cells are not rare; the frequency of the GM was 20.5%. The CD56 and CD49e expressions were significantly higher in the GM group than in the non‐GM group. None of the other potential prognostic factors, including ISS disease stage, M‐protein subtype, and various morphological findings, differed significantly between the GM and non‐GM groups. In this study, GM cells had no significant impact on survival.

## Conflict of Interest

The authors have no potential conflicts of interest to declare.
